# Site-Specific Antibody–Drug Conjugates in Triple Variable Domain Fab Format

**DOI:** 10.3390/biom10050764

**Published:** 2020-05-14

**Authors:** Dobeen Hwang, Christoph Rader

**Affiliations:** Department of Immunology and Microbiology, The Scripps Research Institute, Jupiter, FL 33458, USA

**Keywords:** antibody carrier, catalytic antibody, reactive lysine, antibody engineering, antibody conjugation, cancer therapy

## Abstract

The interest in replacing the conventional immunoglobulin G (IgG) format of monoclonal antibodies (mAbs) and antibody–drug conjugates (ADCs) with alternative antibody and antibody-like scaffolds reflects a need to expand their therapeutic utility and potency while retaining their exquisite specificity, affinity, and low intrinsic toxicity. For example, in the therapy of solid malignancies, the limited tumor tissue penetration and distribution of ADCs in IgG format mitigates a uniform distribution of the cytotoxic payload. Here, we report triple variable domain Fab (TVD–Fab) as a new format that affords the site-specific and stable generation of monovalent ADCs without the Fc domain and a drug-to-antibody ratio (DAR) of 2. TVD–Fabs harbor three variable fragment (Fv) domains: one for tumor targeting and two for the fast, efficient, precise, and stable conjugation of two cargos via uniquely reactive lysine residues. The biochemical and in vitro cytotoxicity properties of a HER2-targeting TVD–Fab before and after conjugation to a tubulin inhibitor were validated. In vivo, the TVD–Fab antibody carrier revealed a circulatory half-life of 13.3 ± 2.5 h and deeper tumor tissue distribution compared to our previously reported dual variable domain (DVD)–IgG1 format. Taken together, the TVD–Fab format merits further investigations as an antibody carrier of site-specific ADCs targeting solid malignancies.

## 1. Introduction

Whereas monoclonal antibody (mAb) therapies as single drug or in combination with systemic chemotherapy have shown limited efficacy in cancer therapy, antibody–drug conjugates (ADCs) are an emerging treatment that maximizes antitumor potency and limits systemic toxicity through the mAb-mediated selective delivery of highly cytotoxic drugs to the tumor [1,2]. Despite their success in the clinic with currently seven Food and Drug Administration (FDA)-approved ADCs for both hematologic and solid malignancies, first-generation ADCs have a suboptimal therapeutic window due to a wide range of drug-to-antibody ratios (DARs; typically 0–8) [3]. This is because first-generation ADCs are assembled by conjugating the payload to surface lysine (Lys) or hinge cysteine (Cys) residues of the immunoglobulin G (IgG; typically IgG1 or IgG4) antibody carrier. This random conjugation creates heterogeneous ADC species with manufacturing, pharmacokinetic, and pharmacodynamic liabilities. To address these shortcomings, numerous site-specific conjugation technologies have been developed to manufacture and administer homogenous ADCs with defined DARs (typically 2 or 4) [4].

Among methodologies affording site-specific ADC assembly, utilizing the uniquely reactive Lys residue (Lys99) of humanized catalytic antibody h38C2 has proven its utility for the fast, efficient, precise, and stable generation of homogeneous ADCs [5]. mAb h38C2 uses the enamine mechanism of natural occurring class Ⅰ aldolases and was developed by reactive immunization of mice with a β-diketone hapten [6,7,8]. In contrast to Lys residues preferentially existing on the protein surface due to the positive charge of the ε-amino group with a typical pKa of 11.0 [9], Lys99 resides at the bottom of an 10-A deep hydrophobic pocket that constitutes the hapten binding site. As such, the ε-amino group of Lys99 has a dramatically perturbed pKa of 6.0; i.e., it is mostly uncharged at physiological pH [7]. The distinctive nucleophilicity of Lys99 enables the hapten-driven selective and covalent conjugation of β-diketone hapten or β-lactam hapten derivatives without labeling other Lys residues [10,11]. Harnessing this unique property of mAb h38C2, we reported a dual variable domain (DVD) IgG1 format [12] composed of an outer variable fragment (Fv) domain targeting tumor cells and an inner Fv domain for site-specific drug conjugation [13]. In the DVD–IgG1 format, h38C2 retains its catalytic activity and Lys99 retains its unique chemical reactivity, enabling the site-specific conjugation of β-lactam hapten derivatized drugs. Forming a stable amide bond, the electrophilic β-lactam hapten group selectively reacts with the nucleophilic ε-amino group of the buried Lys99 residue in each of the two arms of the DVD–IgG1, yielding a DAR of 2. A panel of ADCs built on this DVD–IgG1 format, carrying a β-lactam hapten derivative of monomethylauristatin F (MMAF) and targeting HER2, CD79B, and CD138 revealed subnanomolar and strictly target-dependent cytotoxicity in vitro and, in the case of HER2, highly potent and specific in vivo efficacy [5].

The development of next-generation ADCs has also focused on smaller antibody or antibody-like carriers to enhance tumor mass penetration and tumor cell uptake for the treatment of solid malignancies [14,15]. Conventional antibody carriers in IgG format (approximately 150 kDa) often accumulate around the tumor vasculature and fail to distribute evenly throughout the tumor, resulting in low efficacy and high potential for relapse driven by surviving subpopulations of tumor cells [16,17]. In contrast, smaller antibody or antibody-like carriers may improve tumor penetration, the uptake of ADCs, and the broader distribution of the cytotoxic payload across the tumor tissue. Numerous antibody fragments, such as antigen binding fragment (Fab, approximately 50 kDa), single-chain variable fragment (scFv, approximately 28 kDa), and single-domain antibody (nanobody, approximately 15 kDa), have been utilized for a variety of diagnostic and therapeutic applications [18]. Antibody fragments are more readily able to extravasate into the tumor interstitium and diffuse in the tumor extracellular matrix compared to the larger IgG molecule. Despite these advantages, they may inefficiently accumulate in solid tumors due to rapid systemic clearance by renal excretion and the absence of neonatal Fc receptor (FcRn)-based IgG recycling.

To reduce the molecular weight of the DVD–IgG1 format (approximately 200 kDa) but retain a DAR of 2, we here develop a triple variable domain (TVD)–Fab format (approximately 100 kDa) that uses a tandem inner Fv domain based on mAb h38C2. We show that while ADCs in DVD–IgG1 and TVD–Fab format have similar potency and specificity in vitro, the TVD–Fab penetrates tumor tissue more efficiently and reveals a prolonged circulatory half-life compared to Fab and DVD–Fab.

## 2. Materials and Methods 

### 2.1. Cell Lines

Human breast cancer cell lines SK-BR-3 and MDA-MB-231 were purchased from the ATCC. Human breast cancer cell line KPL-4 was kindly provided by Dr. Naoto T. Ueno based on a Material Transfer Agreement (MTA) with the University of Texas MD Anderson Cancer Center (Houston, TX) and with permission from Dr. Junichi Kurebayashi (Kawasaki Medical School; Kurashiki, Japan). All human breast cancer cell lines were cultured in Dulbecco’s Modified Eagle medium (DMEM) supplemented with 10% (v/v) heat inactivated fetal bovine serum (FBS) and penicillin–streptomycin (containing 100 U/mL penicillin and 100 mg/mL streptomycin; all from Thermo Fisher). Expi293F cells were cultured in Expi293 expression medium supplemented with penicillin–streptomycin (all from Thermo Fisher).

### 2.2. Generation and Conjugation of TVD Fab

The light and heavy chain of the HER2-targeting TVD–Fab encoding sequences consisting of an outer V_L_ or V_H_ domain based on trastuzumab, two inner V_L_ or V_H_ domains based on mAb h38C2, and a C_κ_ or C_H_1 domain were codon optimized and custom synthesized (GenScript). All Fv domains were spaced by an ASTKGP encoding sequence. Following the separate cloning of the TVD light and heavy chains into mammalian expression vector pCEP4 downstream of an N-terminal human CD5 signal peptide (MPMGSLQPLATLYLLGMLVASVLA) encoding sequence via *Xho*Ⅰ and *Nhe*Ⅰ (both NEB) sites, the two plasmids were co-transfected into Expi293F cells at a density of 3 × 10^6^ cells/mL in 300 mL of Expi293 Expression Medium using the ExpiFectamine 293 Transfection Kit (Thermo Fisher). After culturing the transfected Expi293 cells at 37 °C, 5% CO_2_ for 8 days, the culture supernatant was collected and purified by affinity chromatography using a Protein A HiTrap column (GE Healthcare). The eluted protein was brought into 20 mM of 4-(2-hydroxyethyl)-1-piperazineethanesulfonic acid (HEPES, pH 5.5) by dialysis (Slide-A-Lyzer Dialysis Cassettes with a molecular weight cut-off of 10K; Thermo Fisher). After dialysis, 8 μg of the protein was loaded onto a NuPAGE 4–12% Bis-Tris Protein gel (Thermo Fisher), electrophoresed, and stained with Coomassie blue (Thermo Fisher). The yield of TVD–Fab was approximately 5 mg/L as determined by the Pierce BCA Protein Assay Kit (Thermo Fisher). The cloning, expression, and purification of Fab, DVD–Fab, and DVD–IgG1 was described previously [19]. For conjugation, 10 μM of DVD–Fab and TVD–Fab were incubated with 50 μM of β-lactam-hapten-MMAF (compound **1**) [5] or methylsulfone phenyloxadiazole-MMAF (MS-PODA-MMAF; compound **2**) [20] in phosphate-buffered saline (PBS) for 4 h at room temperature (RT). Following incubation, illustra NAP-5 Columns (GE Healthcare) were used to remove free compounds. Then, the ADCs were concentrated with Amicon Ultra 0.5-mL Centrifugal Filters to 1 mg/mL in PBS.

### 2.3. Surface Plasmon Resonance (SPR)

The kinetic and thermodynamic parameters of the binding of TVD–Fab and DVD–Fab to HER2 were analyzed with a Biacore X100 instrument using Biacore reagents and software (GE Healthcare). Following the manufacturer’s instructions, mouse anti-human IgG C_H_2 mAb was immobilized on a CM5 sensor chip using reagents supplied with the Human Antibody Capture Kit (GE Healthcare). Human HER2–Fc fusion protein (R&D Systems) was captured at a density not exceeding 300 RU. Sensor chips included an empty flow cell for instantaneous background depletion. 1x HBS-EP+ running buffer (10 mM HEPES, 150 mM NaCl, 3 mM ethylenediaminetetraacetic acid (EDTA, pH 7.4) and 0.05% (*v*/*v*) Surfactant P20) was used with a flow rate of 30 μL/min. For affinity measurements, all samples were injected at five different concentrations, and the sensor chips were regenerated with 3 M of MgCl_2_ from the Human Antibody Capture Kit. Association (k_on_) and dissociation (k_off_) rate constants were calculated based on a 1:1 Langmuir binding model. The equilibrium dissociation constant (K_D_) was calculated from k_off_/k_on_.

### 2.4. Catalytic Activity Assay

The catalytic activity of mAb h38C2 was analyzed by methodol. After dispensing 1 μM of TVD–Fab and DVD–Fab in 98 μL of PBS into a Corning 96-Well Clear-Bottom Black Polystyrene Microplate (Thermo Fisher) in triplicate, 2 μL of 10 mM methodol was added immediately. Using wavelengths of excitation/emission at 330/452 nm, fluorescence was measured with a SpectraMax M5 instrument (Molecular Devices) in 5-min intervals for 1 h.

### 2.5. Mass Spectrometry

First, 1 mg/ml of TVD–Fab before and after conjugation was prepared for mass spectrometry and data were obtained on an Agilent Electrospray Ionization Time of Flight (ESI-TOF) mass spectrometer. Deconvoluted masses were obtained using Agilent BioConfirm Software.

### 2.6. Cytotoxicity Assay

SK-BR-3 (4 × 10^3^ cells per well) and MDA-MB-231 cells (3 × 10^3^ cells per well) were plated in 96-well tissue culture plates. After adding 10× serially diluted ADCs and their corresponding unconjugated TVD– and DVD–Fab (0.01–100 nM) to the cells, the plates were placed in an incubator at 37 °C in an atmosphere of 5% CO_2_ for 72 h. Cell viability was measured by CellTiter 96 Aqueous One Solution (Promega) following the manufacturer’s instructions and plotted as a percentage of untreated cells. IC_50_ values (mean ± SD) were calculated by GraphPad Prism software. 

### 2.7. Pharmacokinetic (PK) Study

Five female CD-1 mice (approximately 25 g; Charles River Laboratories) per group were tail-vein (i.v.) injected with h38C2 Fab, DVD–Fab, TVD–Fab, or DVD–IgG1 at 6 mg/kg. Post-injection, blood samples were collected at 0, 10 min and 1, 6, 12, 24, 48, 96, and 192 h by tail snipping. After centrifuging the blood at 2000× g for 5 min in a microcentrifuge, the supernatant (plasma) was removed and stored at −80 °C until analysis. The concentration of antibodies in the plasma was measured by ELISA. A Corning 96-well half-area plate (Thermo Fisher) was coated with 100 ng of recombinant human HER2-Fc in 20 μL of carbonate/bicarbonate buffer (pH 9.6) at 4 °C overnight. After blocking with 150 μL 3% (*w*/*v*) bovine serum albumin (BSA)/PBS solution for 1 h at RT, 1:10 or 1:5 PBS-diluted plasma samples were added to the wells and incubated for 2 h at RT. After washing 10 × with 150 μL of 0.05% PBS with Tween 20, 50 μL of 1:5000 diluted peroxidase-conjugated goat anti-human IgG polyclonal antibodies (pAbs; F(ab′)_2_ fragment; Jackson ImmunoResearch Laboratories) in 1% (*w*/*v*) BSA/PBS solution was added to the wells and incubated for 1 h at RT. After developing the plate with 2,2’-azino-bis [3-ethylbenzthiazoline-6-sulfonic acid] (ABTS; Surmodics), the antibody concentration was extrapolated from a four-variable fit of a standard curve. PK parameters were determined with Phoenix WinNonlin PK/PD Modeling and Analysis software (Pharsight) using a two-compartment model. All procedures were approved by the Institutional Animal Care and Use Committee of The Scripps Research Institute and were performed according to the NIH Guide for the Care and Use of Laboratory Animals (PHS Assurance number: D16-00726 (A4460-01)).

### 2.8. Tumor Penetration and Accumulation Study In Vivo

KPL-4 cells (1 × 10^7^ cells per mouse) mixed in an equal volume of BD Matrigel (BD Bioscience) were injected into the mammary fat pad of 7-week-old female NOD scid gamma (NSG) mice (Jackson Laboratory). When the average tumor volume reached 400 mm^3^, tumor-bearing mice received a single intraperitoneal (i.p.) injection of 6 mg/kg of HER-targeting TVD–Fab, HER2-targeting DVD–IgG1, or isotype control (ROR2-targeting) DVD–IgG1. After 24 h, the mice were euthanized by CO_2_ inhalation, and the tumors were removed and dissected. Tumor tissues were equilibrated in a cryoprotective solution containing 30% (*w*/*v*) sucrose in PBS for 24 h at 4 °C followed by fixation in neutral buffer formaldehyde (NBF). The tissue blocks were stored at −80 °C until sectioned. For immunofluorescence staining, cryosections were prepared to a thickness of 4 μm and fixed with NBF buffer for 10 min at RT. After washing in PBS, the sections were blocked with 10% (*v*/*v*) normal goat serum (Sigma-Aldrich) diluted in PBS for 1 h at RT. Next, the sections were incubated with Alexa Fluor 594-conjugated goat anti-human IgG (H+L) pAbs (Invitrogen) for 1 h in a dark and humidified chamber at RT. After again washing the sections in PBS, nuclei were stained with 4′,6-diamidino-2-phenylindole (DAPI; Sigma-Aldrich) according to the manufacturer’s instructions. Finally, the tumor tissue sections were mounted on slides with ProLong Gold Antifade Mountant (Thermo Fisher). Images were obtained at 20× magnification tile scanning and 63× magnification using a Zeiss LSM 880 inverted confocal microscope. All procedures were approved by the Institutional Animal Care and Use Committee of the Scripps Research Institute and were performed according to the NIH Guide for the Care and Use of Laboratory Animals (PHS Assurance number: D16-00726 (A4460-01)).

### 2.9. Size Exclusion Chromatography (SEC)

Unconjugated and MMAF (compound **1**)-conjugated TVD–Fab were analyzed by SEC using a Superdex 200 Increase 10/300 GL column (GE Healthcare) connected to an ÄKTA FPLC system. For each analysis, 30 μL of a 1 mg/mL sample in SEC buffer (50 mM sodium phosphate (pH 7.0) and 150 mM NaCl) were loaded and run. A gel filtration calibration kit for high molecular weight range (GE Healthcare; aldolase (158 kDa), conalbumin (75 kDa), and ovalbumin (44 kDa)) was used as standard. The percentile of aggregates was measured by an integration of peak areas at 280 nm. 

## 3. Results

### 3.1. Generation of TVD–Fab

**Figure 1:**
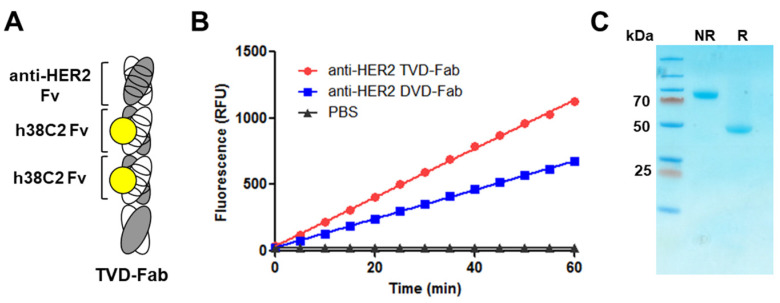
Generation of TVD–Fab. (**A**) TVD–Fab drawing. The depicted TVD–Fab is comprised of an outer HER2-targeting Fv domain and two inner h38C2 Fv domains, followed by constant domains. The reactive Lys99 residue of the V_H_ of h38C2 is marked as yellow circle. (**B**) The catalytic activity of TVD–Fab and DVD–Fab was measured by retro-aldol digestion of methodol to a fluorescent aldehyde (RFU, relative fluorescent units). (**C**) The TVD–Fab was analyzed by SDS-PAGE under non-reducing (NR; approximately 100 kDa) and reducing conditions (R; approximately 50 kDa) followed by Coomassie blue staining. A pre-stained protein ladder (left lane) was used as molecular weight standard.

The concept of doubling the h38C2 Fv domain to create a TVD–Fab with two uniquely reactive Lys99 residues (Figure 1A) relies on the structural integrity of both hydrophobic pockets. Each arm of our original DVD–IgG1-based ADCs consist of a variable outer V_H_/V_L_ (Fv) heterodimer that binds to a tumor antigen and an invariable inner V_H_/V_L_ (Fv) heterodimer derived from mAb h38C2 followed by the C_H_1/C_κ_ heterodimer. In this configuration, the catalytic activity of h38C2 is fully preserved [5]. Several studies have provided evidence for an influence of the C_H_1/C_κ_ heterodimer on the affinity and specificity of antibody–antigen interactions mediated by the V_H_/V_L_ heterodimer [21]. Although mAb h38C2 affords highly homogeneous chemically programmed antibodies and ADCs [5], the extent to which its C_H_1/C_κ_ heterodimer contributes to the integrity of the hydrophobic pocket and thereby affects the reactivity of Lys99 has not been explored experimentally. Since the upper h38C2 Fv domain of the TVD–Fab is connected to the lower h38C2 Fv domain by a short ASTKGP spacer without an intermittent C_H_1/C_κ_ heterodimer (Figure 1A), we first examined the catalytic activity [13] of the tandem h38C2 Fv domains in a HER2-targeting TVD–Fab following its cloning, expression, and purification. Compared to the previously reported DVD–Fab [19], the TVD–Fab revealed approximately 2× enhanced catalytic activity at the same molar concentration (Figure 1B), demonstrating the intact functionality and reactivity of both Lys99 residues. SDS-PAGE under non-reducing and reducing conditions revealed the expected approximately 100-kDa and 50-kDa bands after Coomassie blue staining, confirming the purity and integrity of the TVD–Fab (Figure 1C).

### 3.2. Assembly of TVD–Fab-Based ADCs

**Figure 2:**
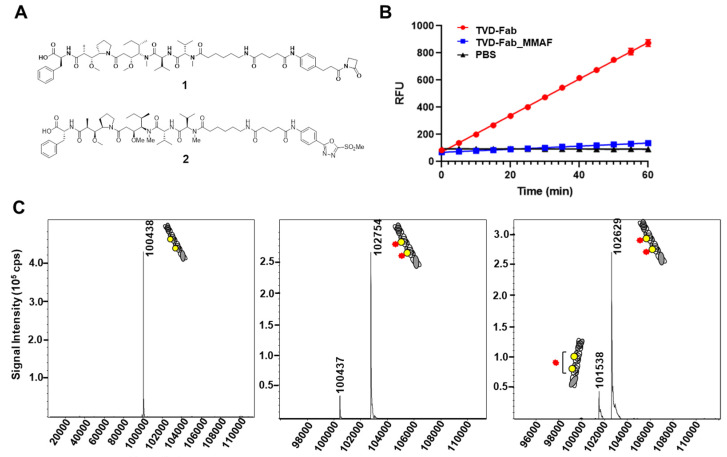
Assembly and analysis of triple variable domain Fab (TVD–Fab)-based antibody–drug conjugates (ADCs). (**A**) Structure of cytotoxic payloads (compound **1**; β-lactam-hapten-MMAF (monomethylauristatin F) and compound **2**; methylsulfone phenyloxadiazole (MS-PODA-MMAF). (**B**) Catalytic activity of TVD–Fab before and after conjugation to compound **1**. (**C**) MALDI-TOF of TVD–Fab before (left) and after conjugation to compound **1** (middle) and compound **2** (right). The MMAF payload is shown as a red star. The expected and observed mass of TVD–Fab was 100,428 Da and 100,438 Da, respectively. After conjugation to compounds **1** and **2**, more than 90% of TVD–Fab was harboring two MMAF payloads with observed masses of 102,754 and 102,629 Da, respectively. The drug-to-antibody ratio (DAR) of TVD–Fab conjugated to compounds **1** and **2** was approximately 1.8 and 1.9, respectively.

Next, we assembled ADCs by incubating the TVD–Fab with five equivalents of β-lactam hapten- or MS-PODA-MMAF (compounds **1**,**2** and Figure 2A). We recently reported that MS-PODA derivatives afford equally fast, efficient, precise, and stable conjugation to Lys99 of h38C2 compared to β-lactam hapten derivatives [20]. Following the removal of unconjugated compounds, the two ADCs were analyzed by the catalytic assay and MALDI-TOF. After conjugation to compound **1**, the TVD–Fab lost its catalytic activity compared to the unconjugated antibody (Figure 2B). MALDI-TOF revealed an observed mass of the unconjugated TVD–Fab of 100,438 Da (expected mass 100,427 Da; Figure 2C, left). The major (>90%) and minor (<10%) peaks of the conjugated TVD–Fab revealed masses of 102,754 Da and 100,437 Da, corresponding to DARs of 2 (expected mass 102,749 Da) and 0 (expected mass 100,438 Da; Figure 2C, middle). Interestingly, no DAR of 1 species was detected. Similar to the TVD–Fab _**1** ADC, >90% of TVD–Fab conjugated to compound **2** had a DAR of 2 with an observed mass of 102,629 Da (expected mass 102,659). The remaining portion of the TVD–Fab _**2** ADC had a DAR of 1 (observed and expected mass of 101,538 Da and 101,543 Da respectively; Figure 2C, right).

### 3.3. Affinity and Activity of TVD–Fab-Based ADCs

**Figure 3:**
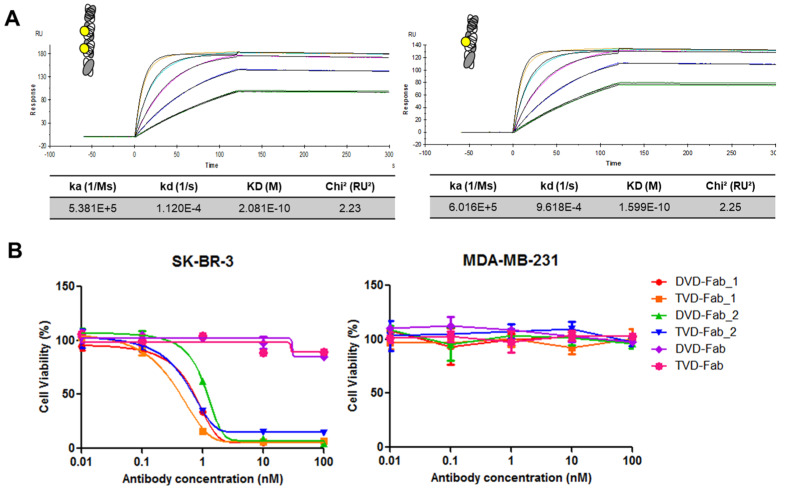
Affinity of anti-HER2 TVD–Fab and dual variable domain (DVD)–Fab and in vitro cytotoxicity of the corresponding antibody–drug conjugates (ADCs). (**A**) Surface plasmon resonance (SPR) analysis of the binding of TVD–Fab and DVD–Fab to immobilized recombinant HER2–Fc fusion protein. (**B**) Cytotoxicity of the TVD–Fab and DVD–Fab before and after conjugation to β-lactam-hapten-MMAF (**1**) or MS-PODA-MMAF (**2**). The IC_50_ of TVD–Fab- and DVD–Fab-based ADCs were 0.32 and 1.6 nM (**1**), and 3.2 and 10 nM (**2**) against HER2-positive SK-BR-3 cells without showing cytotoxicity against HER2-negative MDA-MB-231 cells.

The affinity of TVD–Fab and DVD–Fab for recombinant HER2–Fc fusion protein was measured by SPR, revealing K_D_ values of 2.1 × 10^−10^ M and 1.6 × 10^−10^ M, respectively (Figure 3A). Next, the in vitro cytotoxicity of the TVD–Fab and DVD–Fab-based ADCs against HER2-positive (SK-BR-3) and HER2-negative (MDA-MB-231) breast cancer cell lines was compared. TVD-Fab_**1**, TVD-Fab_**2**, DVD-Fab_**1**, and DVD-Fab_**2** killed SK-BR-3 cells at subnanomolar to low nanomolar concentrations. By contrast, MDA-MB-231 cells remained alive at 100 nM, which was the highest concentration tested. As expected from their higher DAR, both TVD–Fab-based ADCs revealed lower IC_50_ values than their corresponding DVD–Fab-based ADCs (Figure 3B). 

### 3.4. Monodispersity of TVD–Fab and TVD–Fab-Based ADC

**Figure 4:**
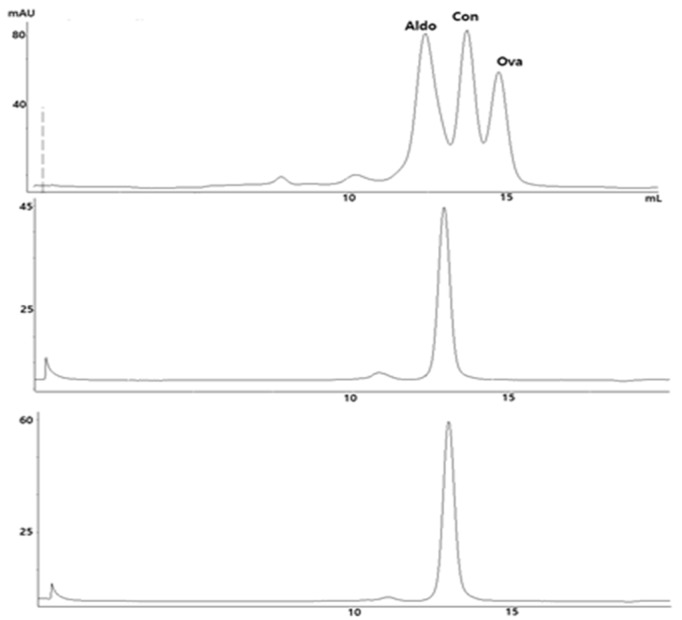
Size exclusion chromatography (SEC) analysis of TVD–Fab before and after conjugation to β-lactam-MMAF (**1**). Aldolase (Aldo; 158 kDa), conalbumin (Con; 75 kDa), and ovalbumin (Ova; 44 kDa) were used as molecular weight standard (top).

The monodispersity of the TVD–Fab before and after conjugation to β-lactam hapten-MMAF (**1**) was analyzed by SEC using a molecular weight standard for comparison. Monodisperse TVD–Fab and TVD–Fab_MMAF peaks (approximately 100 kDa) appeared between the peaks of aldolase (158 kDa) and conalbumin (75 kDa). Preceding minor peaks indicating aggregation corresponded to 5.6% (TVD–Fab) and 2.6% (TVD–Fab_MMAF) of total protein (Figure 4).

### 3.5. PK Properties of TVD–Fab

**Figure 5:**
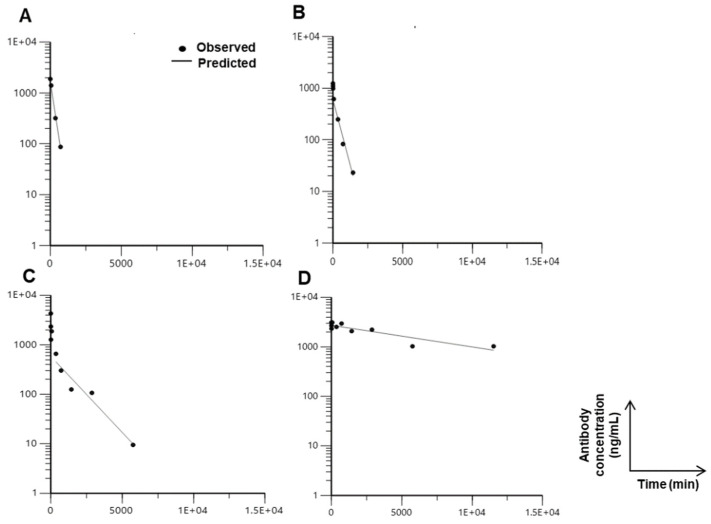
Pharmacokinetic (PK) profiling of antibody carriers in various formats. Five female CD-1 mice each per group were intravenously (i.v.) injected with 6 mg/kg of h38C2 Fab (**A**), anti-HER2 DVD–Fab (**B**), anti-HER2 TVD–Fab (**C**), and anti-HER2 DVD–IgG1 (**D**). Peripheral blood samples were collected at 5 and 10 min, 1, 6, 12, 24, 48, 96, and 192 h after injection, and used for ELISA to calculate the concentration of antibody carrier in each sample. By using Phoenix WinNonlin PK/PD Modeling and Analysis software, the circulatory half-life (t_½_) of antibody carriers was determined. The plots show one representative mouse from each group. All plots show the typical biphasic graph of fast dissemination followed by slow secretion. A two-compartment model was used to determine the t_½_ of h38C2 Fab (2.2 ± 0.3 h), anti-HER2 DVD–Fab (5.4 ± 0.5 h), anti-HER2 TVD–Fab antibody (13.3 ± 2.5 h), and anti-HER2 DVD–IgG1 (124 ± 12.5 h).

The larger molecular weight of the TVD–Fab (approximately 100 kDa) predicted a longer circulatory half-life compared to DVD–Fab (approximately 75 kDa) and Fab (approximately 50 kDa) but substantially shorter than DVD–IgG1 due to the lack of an Fc domain enabling neonatal Fc receptor (FcRn)-mediated recycling. This was investigated in a PK study for which anti-HER2 TVD–Fab, DVD–Fab, h38C2 Fab, and DVD–IgG1 were each injected intravenously (i.v.) into five CD-1 mice, and plasma prepared from various time points was analyzed by ELISA to determine the antibody carrier concentration (Figure 5). As anticipated, DVD–IgG1 and h38C2 Fab showed the longest and shortest circulatory half-lives with 124 ± 12.5 h and 2.2 ± 0.3 h, respectively. Notably, the TVD–Fab revealed a >2× longer circulatory half-life (13.3 ± 2.5 h) compared to the DVD–Fab (5.4 ± 0.5 h).

### 3.6. Tumor Tissue Penetration and Distribution of TVD–Fab versus DVD–IgG1

**Figure 6:**
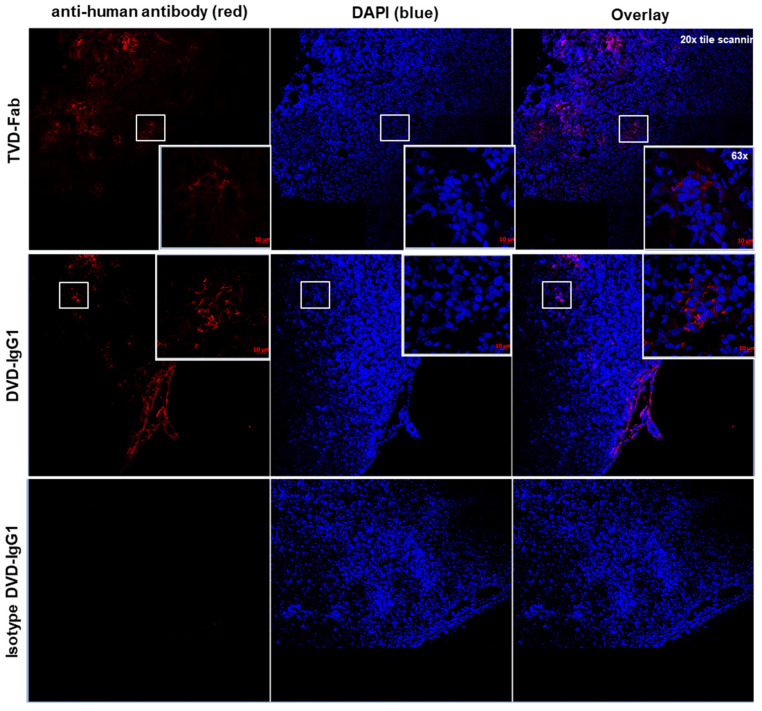
Tumor tissue penetration and distribution of anti-HER2 TVD–Fab and DVD–IgG1 in an orthotopic mouse xenograft model of breast cancer. Representative images from confocal microscopy. Human KPL-4 cells were injected into the mammary fat pad of 7-week-old female NOD scid gamma (NSG) mice. When tumors reached 400 mm^3^, the mice were intraperitoneally (i.p.) injected with 6 mg/kg of anti-HER2 TVD–Fab, anti-HER2 DVD–IgG1, and an isotype control DVD–IgG1. After 24 h, tumor tissue penetration and distribution of the human antibodies was visualized. Image magnification, 20× and 63×.

Since the TVD–Fab antibody carrier had a relatively long circulatory half-life for an antibody carrier without Fc domain and its size (approximately 100 kDa) falls between IgG1 (approximately 150 kDa) and Fab (approximately 50 kDa), we hypothesized that it may more efficiently penetrate into and accumulate in tumor tissue compared to the DVD–IgG1 antibody carrier (approximately 200 kDa). To test this, we used an established orthotopic xenograft (approximately 400 mm^3^) of human breast cancer cell line KPL-4, which expresses high levels of HER2 [22], in immunodeficient mice. At 24-h post-injection of anti-HER2 TVD–Fab, DVD–IgG1, and an isotype control DVD–IgG1, the mice were euthanized to remove and dissect their tumors. Then, blocks of tumor tissue were processed to monitor the distribution of the antibody carriers. For this, antibody carriers were stained with Alexa Fluor 594-conjugated goat anti-human IgG (H+L) pAbs (red), and nuclei were stained with DAPI (blue). As shown in Figure 6, most of the HER2-targeting DVD–IgG1 was observed in the marginal area surrounding the tumor. In clear contrast, the TVD–Fab was primarily detected inside the tumor tissue. The isotype control DVD–IgG1 was not detected outside or inside the tumor tissue.

## 4. Discussion

ADCs are comprised of three principal components with distinct roles: (i) the antibody carrier binds to an internalizing tumor antigen with high specificity and affinity; (ii) the linker is designed to provide stable attachment of the cytotoxic payload to the antibody carrier in circulation and to permit its release inside the cell; and (iii) the cytotoxic payload kills tumor cells at subnanomolar to low nanomolar concentrations. Each component has been researched extensively to develop and optimize pharmaceutically active ADCs for cancer therapy [23]. Furthermore, the site-specific assembly of the three components to afford homogeneous ADCs with uniform DARs is anticipated to result in improved therapeutic efficacy, reduced systemic toxicity, and preferable PK properties, as has been established by comparing heterogeneous and homogeneous ADC assemblies [24].

The movement of ADCs from blood to tissue is proceeding by convection relying on the blood–tissue hydrostatic gradient and paracellular pores in the vascular epithelium, passive diffusion, or receptor-mediated endocytosis [25]. However, the anatomical and physiological properties of solid tumors prevent efficient tumor tissue penetration and distribution of ADCs. In fact, experiments with radiolabeled antibodies in IgG format show that only approximately 0.01% of the injected dose accumulates in the tumor tissue [26,27]. To improve tumor penetration, distribution, and accumulation of mAbs and ADCs in solid malignancies, a variety of smaller antibody carriers compared to the conventional IgG format have been developed [18,28]. However, it was found that elimination by the kidney through glomerular filtration and the ensuing short circulatory half-life mitigates efficient tumor tissue accumulation of antibody carriers with molecular weights <50 kDa.

We previously developed a new site-specific ADC platform that uses an antibody carrier in DVD–IgG1 format [5]. While this antibody carrier permitted fast, efficient, precise, and stable conjugation of two cytotoxic payloads via uniquely reactive Lys residues for a DAR of 2, its large molecular weight (approximately 200 kDa) was considered a potential liability for the treatment of solid malignancies. We here report the development of a TVD–Fab format (approximately 100 kDa) that preserves all biochemical properties of the DVD–IgG1 format including a DAR of 2 but cuts the molecular weight in half. The HER2-targeting TVD–Fab prototype we built and tested was produced with acceptable yields and revealed little aggregation before and after conjugation.

Due to the lower molecular weight and primarily due to the absence of an Fc domain that mediates FcRn recycling, the circulatory half-life of TVD–Fab (approximately ½ day in mice) is significantly shorter than that of DVD–IgG1 and may require every-other-day (qod) or twice-weekly (biw) dosing in humans. As ADCs typically reveal increased clearance compared to mAbs [29], we expect the circulatory half-life of TVD–Fab-based ADCs to be shorter than their TVD–Fab antibody carriers. Nonetheless, we showed that a HER2-targeting TVD–Fab penetrated a HER2-expressing orthotopic breast cancer xenograft more efficiently than its DVD–IgG1 counterpart when tested one day after administration, suggesting that tumor tissue accumulation can be achieved with a qod or biw dosing regimen. The absence of an Fc domain can overcome the Fc-related toxicity of conventional ADCs caused by their uptake and degradation by Fcγ receptor-expressing macrophages in healthy tissues, such as Kupffer cells in the liver and alveolar macrophages in the lung [30]. Another Fc-related toxicity was suggested to be driven by the carbohydrates on the Fc domain, specifically their recognition by the mannose receptor [30,31]. The aglycosylated TVD–Fab evades both Fcγ receptor and mannose receptor-mediated uptake that can lead to the off-target toxicity of ADCs. Collectively, the TVD–Fab format has a unique pharmacodynamic and pharmacokinetic profile that warrants further evaluation in in vivo models of solid tumors.

The modular composition of the TVD–Fab with a variable outer Fv targeting a tumor antigen and an invariable inner tandem Fv for the site-specific conjugation of two identical cargos via β-lactam hapten or MS-PODA conjugation chemistries enables broad therapeutic and diagnostic utility in and beyond cancer. Furthermore, the TVD–Fab format can be adapted to carry two different payloads by substituting one of the reactive Lys residues with an arginine (Arg) residue, enabling orthogonal one-pot and one-step conjugation to afford Lys99:β-lactam hapten and Arg99:phenylglyoxal adducts [19]. For applications that do not rely on tissue penetration and distribution, converting the TVD–Fab to a TVD–IgG1 (approximately 250 kDa) with either four reactive Lys residues or a combination of two reactive Lys and two reactive Arg residues provides another attractive modality.

## 5. Conclusions

Our study supports the therapeutic utility of the TVD–Fab format for the generation of site-specific ADCs with a DAR of 2 and a relatively long circulatory half-life for an antibody carrier without an Fc domain. Further preclinical studies to find a dosing regimen for the optimal tumor tissue accumulation of the cytotoxic payload delivered by the TVD–Fab and for a direct comparison of the therapeutic indices of TVD–Fab and DVD–IgG1 are warranted.
